# Do “Girls Just Wanna Have Fun?” Participation Trends and Motivational Profiles of Women in the Birkebeiner Races, Norway’s Ultimate Mass Participation Ski Event

**DOI:** 10.3389/fpsyg.2019.02548

**Published:** 2019-11-26

**Authors:** Giovanna Calogiuri, Patrick Foss Johansen, Alessio Rossi, Miranda Thurston

**Affiliations:** ^1^Section for Public Health, Department of Public Health and Sport Sciences, Faculty of Social and Health Sciences, Inland Norway University of Applied Sciences, Elverum, Norway; ^2^Department of Computer Science, University of Pisa, Pisa, Italy

**Keywords:** cross-country skiing, machine learning, mass participation sport, physical activity, women’s health

## Abstract

Mass participation sporting events (MPSEs) are viewed as encouraging regular exercise in the population, but concerns have been expressed about the extent to which they are inclusive for women. This study focuses on an iconic cross-country skiing MPSE in Norway, the Birkebeiner race (BR), which includes different variants (main, Friday, half-distance, and women-only races). In order to shed light on women’s participation in this specific MPSE, as well as add to the understanding of women’s MPSEs participation in general, this study was set up to: (i) analyze trends in women’s participation, (ii) examine the characteristics, and (iii) identify key factors characterizing the motivational profile of women in different BR races, with emphasis on the full-distance vs. the women-only races. Entries in the different races throughout the period 1996–2018 were analyzed using an autoregressive model. Information on women’s sociodemographic characteristics, sport and exercise participation, and a range of psychological variables (motives, perceptions, overall satisfaction, and future participation intention) were extracted from a market survey and analyzed using a machine learning (ML) approach (*n* = 1,149). Additionally, qualitative information generated through open-ended questions was analyzed thematically (*n* = 116). The relative prevalence of women in the main BR was generally low (< 20%). While the other variants contributed to boosting women’s participation in the overall event, a future increment of women in the main BR was predicted, with women’s ratings possibly matching the men’s by the year 2034. Across all races, most of the women were physically active, of medium-high income, and living in the most urbanized region of Norway. Satisfaction and future participation intention were relatively high, especially among the participants in the women-only races. “Exercise goal” was the predominant participation motive. The participants in women-only races assigned greater importance to social aspects, and perceived the race as a tradition, whereas those in the full-distance races were younger and gave more importance to performance aspects. These findings corroborate known trends and challenges in MPSE participation, but also contribute to greater understanding in this under-researched field. Further research is needed in order to gain more knowledge on how to foster women’s participation in MPSEs.

## Introduction

### Mass-Participation Sporting Events, a Vehicle for Health Promotion?

Mass participation sporting events (MPSEs) have been defined as sporting competitions “where the primary focus is on promoting participation and engagement rather than the significance of the sporting outcome” (Coleman and Ramchandani, 2010). In contrast with other popular sporting events that receive large coverage in the media, such as the Olympic Games and different world championships, a characteristic of MPSE is that they not only attract elite athletes but, in the spirit of “sport for all” ideals, they primarily target recreational sport practitioners. This, alongside the fact that MPSEs have experienced a large increase in popularity across the world in the last three decades, means that MPSEs have been increasingly viewed as a possible vehicle for health promotion (Murphy and Bauman, 2007; Stevinson and Hickson, 2013; Murphy et al., 2015). In particular, MPSEs can provide a motivational goal for people who intend to start exercising regularly or for those who want to enhance their exercise routines.

Some research evidence suggests that MPSEs might indeed be effective in encouraging some people to enhance their exercise habits. For example, Bowles et al. (2006) found an increased frequency of biking sessions post-event compared with pre-event among first-time participants in a mass-participation biking event in Sydney. Funk et al. (2011) argue that MPSEs might also encourage lifelong patterns of sports participation, an issue that is particularly important given the tendency for physical activity levels to decline as people age, a trend that is especially common among women (McArthur et al., 2014). Moreover, MPSEs can have a social value in that the community-oriented and celebratory nature of events can generate feelings of satisfaction and inspiration that might be important in locking people into participation (Chalip, 2006). Discussing physical activity participation more generally, Gough et al. (2018) argue that there is “a web of influences” beyond individual-level accounts that relate to the context. This suggests that the context of MPSEs and the kinds of experiences they might generate is important in understanding participation.

It should, however, be noted that to use sport as an effective vehicle for health promotion, it is essential to focus on narrowing the social gradient that characterizes sport participation. In particular, promoting sport and exercise participation in those groups that tend to be less “sporty” (e.g., women, older people, and those with low socio-economic status) has been viewed as a key strategy for improving population health (World Health Organization [WHO], 2018). In this regard, concerns about the extent to which MPSEs attract those from social groups of relevance to public health have been raised (Murphy and Bauman, 2007; Murphy et al., 2015), although it has been reported that *some* events, especially those characterized by less challenging trails and a less competitive atmosphere (e.g., the Women’s Mini Marathon in Ireland), were more successful in attracting individuals with low or moderate levels of physical activity (Bauman et al., 2009). Notwithstanding the convergence in performance between men and women in recent years in specific events (see, e.g., Hausken, 2019), from a public health perspective offering variant events (such as shorter distance and women-only races) may be beneficial if those from underrepresented groups (such as relatively inexperienced women) are to be drawn in.

### Understanding Why People Participate in Mass-Participation Sporting Events

When considering the issue of exercise promotion, especially in relation to encouraging people to increase their physical activity levels and/or maintain high physical activity levels over time, understanding people’s motives is central. Knowledge on what motivates people to start and continue exercising, or more specifically what motivates them to participate in an MPSE, can inform promotional campaigns targeting specific groups of individuals. The act of purposefully training for and participating in an MPSE can be qualified as a goal-directed behavior (Forster et al., 2007). It is important to note, however, that different participants may perform such goal-directed behavior for varying reasons (Funk et al., 2011).

In general, studies have previously shown that participation in sports tends to be driven by different motivations compared to other forms of exercise. In particular, participation in sporting activities was found to be predominantly driven by enjoyment and mastery motives, while participating in fitness activities and other exercise forms was found to be primarily driven by appearance-related motives (Frederick and Ryan, 1993; Kilpatrick et al., 2005). Moreover, participation in organized sports has been associated with sociability motives to a larger extent than, for example, participation in gym-based and nature-based exercise such as walking or running in natural environments, the latter being more strongly associated with convenience-related motives as well as the enjoyment of being in contact with nature (Calogiuri and Elliott, 2017). More specifically in the context of MPSEs, a study among road running participants in the United States identified two major tiers depicting the participants’ motivational profiles (Funk et al., 2011). A first, more common tier (reflected in 95% of the study participants), consisted of a combination of motives related to “challenge,” “enjoyment,” “strength and endurance,” and “positive health.” The second tier, less common but still largely prevalent (reflected in 75% of the participants), consisted in the combination of motives related to “competition,” “weight management,” “ill-health avoidance,” “social affiliation,” “physical appearance,” and “stress management.” Noticeably, both these tiers of motives were significantly related to the participants’ commitment to running and future exercise intention.

In light of these findings, it is plausible to postulate that the issue of the participation motive for MPSEs is complex. Moreover, different events (and sub-events) are likely to attract participants with different performance level as well as different motivational profiles. On the other hand, individuals’ motivation does not represent the only factor that explains sport participation, which is, as previously noted, often subject to a social gradient. For example, a review by Murphy et al. (2015) reports that, among MPSEs’ participants, women, ethnic minorities, and people with lower previous physical activity levels are often under-represented. Moreover, a systematic review of 131 studies by Beenackers et al. (2012) found consistent evidence of the association between individuals’ socio-economic status and participation in leisure-time physical activity, with the most consistent socio-economic inequalities found for vigorous activities such as sports.

### Women in Mass-Participation Sporting Events

Globally, women have lower physical activity levels than their male counterparts (World Health Organization [WHO], 2010, 2018; Hallal et al., 2012). Over the past few decades, however, women’s physical activity levels in many high-income countries have increased. In United Kingdom, for example, the prevalence of women meeting the minimum recommendations for physical activity is almost equal to men’s (The House of Commons [THoC], 2017) and in Norway, the prevalence of sufficiently active women is slightly higher than that of men (Norwegian Directorate of Health, 2015). While women’s high levels of physical activity are generally mainly driven by activities such as walking and exercising in fitness centers, their participation in other sporting activities such as running and biking tends to be lower compared to men (THoC, Statistics Norway, s.a.; 2017). In line with this pattern, in Norway, women’s participation in cross-country skiing is relatively high, though lower than that of their male counterparts (Statistics Norway, s.a.).

In line with these trends, women tend to be under-represented in MPSEs (Murphy et al., 2015). A study of the characteristics of the approximately 10,000 participants in Sydney’s annual Spring Cycle showed that less than 30% of them were women (Bowles et al., 2006). These figures are in line with a recent analysis of 2,195,588 recreational marathon runners across different countries, which found that, on average, women made up only 30% of total participants (Andersen, 2019). There are, however, some signs that MPSEs are, indeed, becoming increasingly attractive for women. For instance, the analyses of recreational marathon runners showed that in the past 10 years, the growth of women’s participation was threefold compared with that of men’s, i.e., 27% and 8%, respectively (Andersen, 2019). As a matter of fact, in some MPSE (especially running events in Northern America) women’s participation ratings already match that of men. For example, in a survey conducted among road race participants in the United States, Funk et al. (2011) found that women made up 55% of overall participants, a finding that is in line with commercial reports (see, e.g., Running USA, 2014).

To boost women’s participation specifically, many MPSEs offer women-only races, which typically take place on shorter and/or less challenging routes and are characterized by a less competitive atmosphere. Some studies suggest that women-only races not only catalyze women’s participation in MPSEs, they also may help them maintain high physical activity levels over the years (Crofts et al., 2012; McArthur et al., 2014). While the addition of women-only races might be effective in boosting women’s participation in absolute terms, enhancing the inclusiveness of women in main MPSEs may also have advantages, such as contributing to reducing gender stereotypes in sport as well as stimulating even greater amounts of exercise among female participants. Moreover, these events are likely to attract women who have different characteristics, motives, and aspirations than those who would rather enter main MPSE. In particular, it is plausible to assume that women in the main MPSEs have higher levels of physical training and are more interested in their performance, as opposed to the participants in women-only races, who may attach more importance to the social context and supportive and celebratory atmosphere of the event.

To date, there is a dearth of scientific studies and academic publications specifically investigating women’s participation in MPSEs, both in terms of participation trends as well as the characteristics, motives, and aspirations of the women who attend. Furthermore, little is known about how such characteristics, motives, and aspirations may differ in different race contexts, especially with respect to female participants in *main* MPSEs or *women-only* races. Furthermore, it should be noted that the issue of women’s inclusion in MPSEs is complex and presents a number of challenges. For example, it is argued that this phenomenon primarily benefits women who are already active and those from more advantaged social groups, while inactive women from more disadvantaged groups (e.g., older women, those from ethnic minorities and with lower socioeconomic status) tend to remain excluded (Murphy et al., 2015). A better understanding of this complex phenomenon is needed in order to assist initiatives in fostering a culture in MPSEs that encourages the participation of women from across these under-represented groups.

### The Birkebeiner Races

In the present study, we focus on a particular MPSE, the Birkebeiner races (BRs). The BRs are an iconic Norwegian cross-country ski (classic technique) MPSE, which takes place in the region of Oppland (Inland Norway) and registers over 10,000 participants every year. The challenging 54 km trail goes through open and forest terrains, crossing two mountains (820 and 760 m above sea level). In 2018, the main BR celebrated its 80th edition; the race was launched for the first time in 1932, and since then it has been organized annually, except in the war years 1941–1945 and few other times because of adverse meteorological conditions. Alongside the main race, the event includes different variants: the Friday, the half-distance, and the women-only races. The Friday race takes place on the exact same trail two days before the main BR, and is generally characterized by fewer participants attending. Differently, the half- (28 km) and the women-only (15and 30 km) races are characterized by shorter and less challenging (relatively to the main BR) tracks. For instance, the track of the women-only 15 km race has only one major up-hill (about 550 m above sea level).

The BRs have received some research attention, especially in relation to its economic impact in the region (Stevik, 2008) as well as in relation to perspectives in sport management (Slåtten et al., 2014), sport medicine (Myrstad et al., 2014), and traumatology (see, e.g., Butcher and Brannen, 1998). On the other hand, less attention has been given to this particular event in relation to the participants’ characteristics and motives, and the existing information on this particular perspective is mainly available through market reports produced by the race organizers or popular-scientific publications. In line with international literature on MPSEs (Murphy and Bauman, 2007; Murphy et al., 2015), a 2011 survey among about 900 participants in the main BR found that most lived in larger cities, were highly educated, well trained, and of male sex; women made only 19% of total participants (Rønning and Skaare, 2011). “Health” was the most common participation motive, which is generally in line with research on motives for physical activity in the Norwegian population (Norwegian Directorate of Health, 2015) as well as other Scandinavian populations (see, e.g., Aaltonen et al., 2014). Seeing the race as a personal challenge (which indicates *intrinsically* regulated motivation) was also a commonly reported participation motive. Remarkably, this motive was found to be more prevalent among men compared to women.

### Purpose of the Present Study

Norway boasts relatively high sports participation rates, compared to countries beyond the Nordic region, including among women (Dalen and Holbaek-Hanssen, 2016; Green et al., 2018). This provides an interesting context to study the phenomenon of women’s participation in MPSEs. Moreover, the BRs are also interesting with respect to its particular location, as the region in which it takes place (Inland Norway) has one of the worst public health profiles in Norway, including having the lowest levels of physical activity compared with the rest of the country. Specifically, in order to shed light on women’s participation in the different variants of the BR event, as well as contribute knowledge of women’s participation in MPSEs in general, the purpose of this study was threefold:

I Analyze trends in women’s participation in the different variants of the BRs;
II descriptively examine the characteristics of women participating in different variants of the event, with an emphasis on the full-distance compared to the women-only races, in terms of: sociodemographic profile, sport and exercise participation, and a range of psychological factors (motives, self- and race-perceptions, overall satisfaction, and future participation intention); and
III by triangulating findings generated through a machine learning (ML) approach and a thematic analysis, identify key factors characterizing the motivational profile of women participating in either the full-distance or the women-only races.

## Materials and Methods

### Women’s Participation Trends

#### Design and Data

In order to analyze trends in women’s participation in the different variants of the BR event (objective I), a time-series analysis of their participation rates based on entry records was undertaken. Entry records of both men and women were provided by the event organizers (Birken AS), referring to the number of participants who, in each year, signed up for the different races. Data on starters (those who actually participated in the race) were also examined but not presented in this paper as the difference between these and the starting participants was fairly constant throughout the years. For the main BR, women’s entries were available for the period of 1996–2018. For the Friday, the half-distance, and the women-only races (the latter, separately for the 15 and 30 km distances), entry records were obtained from the first year the races were introduced, i.e., 2010, 2012, and 2013, respectively.

#### Analyses

Women’s entry records were examined both as absolute numbers and as relative prevalence of women participants with respect to the overall number of participants. Autoregressive models (Wei, 2013) were created in order to forecast future participation in the main BR. The autoregressive approach allows the prediction of an event based on a weighted sum of past values reflecting the secular trend. Three time-series were analyzed for the period 1996–2018: total participants, relative prevalence of men, and relative prevalence of women. For each time series, an exponential weighted moving average (EWMA) with 6 years span was computed in order to avoid extreme frequency values that could affect the prediction of future trends. The model was trained on *n*-10 elements of the time series (i.e., 1996–2008) and tested on the remaining 10 waves (i.e., 2008–2018). The accuracy of the model was assessed by computing the mean squared error (MSE) between the observed and predicted values. Finally, the year in which the relative prevalence of women in the race was predicted to be equal to that of men’s was based on the autoregressive function extracted from the participation frequency recorded between 1996 and 2009.

Because of the small number of observations (i.e., waves available for the analysis), it was not possible to apply the autoregression on the other races (Friday, half-distance, and women-only races). The participation trends for Friday, the half-distance, and the women-only races where thus only examined through descriptive statistics.

### Characteristics of Women Participating

#### Design and Participants

In order to examine the characteristics of women participating in the different variants of the BR event (objective II), as well as identify distinctive characteristics of women participating in the full-distance vs. the women-only race (objective III), a secondary analysis of data based on a market survey conducted by the race organizers was carried out. In order to perform a trend analysis on the participants’ characteristics (similar to that which was done with the participation ratings), we examined the series of surveys from previous years. Although the race organizers had been conducting market surveys for several years (to our knowledge, starting from 2012), they have significantly changed the questionnaire over time, as well as across the different events, with only relatively few items being consistent. In particular, starting from 2016, the race organizers changed the statistical agency that planned and delivered the survey, leading to major changes in the structure and quantity of items included. This particular survey wave had a high degree of consistency across the different races, with the exception of the half-distance race (for which a very different questionnaire, designed directly by the race organizers, was used). We have no knowledge of other survey waves being carried out after 2016. Based on this preliminary examination, it was decided to only include the survey wave from 2016 in our analyses.

The survey was administered online by an independent market research company (Differ Strategy Consulting, Oslo) about 1 month after completion of the races among participants in the main BR, Friday, and the women-only races. The race organizers performed a separate survey among the participants of the half-distance race, but because the questionnaire used was substantially different from the one used for the other races, this information was not included in the present study. All people registered for the different races were invited to participate in the survey. Only data from female respondents who confirmed their participation in either the main BR, the Friday, and the women-only races were used for the analyses (overall *n* = 1,187, overall response rate 35%). Details about the respondents and response rates for each individual race are presented in Table 1. The use of the data was approved by the Norwegian Centre for Research Data (Project No. 58439).

**TABLE 1:** Race characteristics and overview of women participants and respondents to a market survey in 2016^a^.

**Race**	**Distance**	**Participants**	**Respondents**	**Response rate**
	**(km)**	**(*N*)**	**(*n*)**	**(%)^b^**
Main event	54	1546	515	33
Friday event	54	306	97	31
Half-distance^c^	28	239	96	40
Women-only 15 km	15	1,054	378	36
Women-only 30 km	30	494	197	40
All		3,639	1,283	35

*^*a*^Based on official records available online (http://historikk.birkebeiner.no/index2.php) cross-matched with records by Birken AS. ^*b*^The response rate was calculated on the total *N* of participants, who were all invited to participate in the survey. ^*c*^Data from the market survey on the half-distance race were not included in the analyses.*

#### Measures

The market survey included, among other things, a set of items that were primarily designed for commercial purposes (e.g., questions regarding the brand of equipment used and visibility of sponsors). These items were removed, while only items relative to the variables of interest for the study purpose were retained, based on the assumption that they were found in identical or fairly similar form across the different races. If necessary, the items were recoded to improve the consistency across the different races, or to better address the purpose of the study (see description of variables below). These items included three groups of variables: sociodemographic characteristics, sport and exercise participation, and psychological variables (motives for participating in the race, self-perception and perceptions of the race, overall satisfaction with the race, and future participation intention). Additionally, qualitative information (responses to open-ended questions) relative to the women’s motivations and perceptions were also extracted for further analyses.

##### Sociodemographic characteristics

This included age (measured as a continuous variable), income (1 = < 200.000 NOK, 2 = 200.– 400. NOK, 3 = 400.–600 NOK, 4 = 600.–800 NOK, 5 = > 800. NOK), and region of residence. The latter was originally measured by selecting either one of the 20 Norwegian counties or the option “abroad.” These were recoded into four categories: “host region” (Inland Norway, i.e., the region that hosts the races); “adjacent, most urbanized” (i.e., Oslo and Akershus; the most densely populated region, which is well connected by road and rail to the host region); “other adjacent regions” (South-Eastern Norway, the southern parts of Central Norway, which are relatively close to the host region); “farther regions” (South-Western Norway, Western Norway, the Northern parts of central Norway, Northern Norway, and abroad).

##### Sport and exercise participation

“Other races” was a dichotomous variable indicating whether or not the women usually participated in other sporting events, of small or large scale, either in Norway or abroad. “Ski-based exercise” was a categorical variable indicating the women’s ski-based exercise levels compared with the past 5 years (“Compared with 5 years ago, do you engage in ski-based exercise …”: 1 = In smaller amounts, 2 = More or less in equal amounts; 3 = In larger amounts). “Sum of sports” provided an indication of the variety of exercise activities in which the women were planning to engage in the spring season, and was constructed based on a multiple-choice item inquiring “What will be your main exercise activity in the next months?,” to which the respondents answered by selecting one or more (or none) of six listed options: running, biking, roller-ski, team-sports, strength exercise, other. The individual exercise options were included in the analyses as dummy variables (Option selected = 0; Option non-selected = 1), and an additional variable was derived by summing the number of options selected, and ranged from 0 to 6.

##### Psychological variables

Eight different motives (e.g., “To measure myself against friends” and “I usually participate every time”) were measured as dummy variables by asking the women to report whether or not they considered each particular motive as an important reason to enter the race. The women’s self-perception during the race (seven items, such as “Challenged” and “Part of a community”) and perceptions of the race (seven items, such as “Nice experience” and “Nice social setting”) were measured on a 1–6 scale (1 = “It suits me very little”; 6 = “It suits me very well”). The women’s overall satisfaction with the race was measured using a 1–10 scale (1 = “Absolutely dissatisfied”; 10 = “Absolutely satisfied”). Future participation intention was a dummy variable with 1 corresponding to the high intention to participate again the following year (i.e., “Extremely likely”) while 0 corresponded to any lower intention.

##### Qualitative data

Qualitative information was collected through an optional open-ended question that allowed the participants to comment on their own motives for participating and perceptions regarding the race. This question asked: “what would you say was your main motivation for participating in the race?” Among the sample, qualitative data were available from 116 women in total (note: the open-ended items were not compulsory and respondents could choose whether or not provide more in depth information in these sections).

#### Analyses

##### Sample representativeness

In an attempt to estimate the extent to which the sample of respondents (*n*) was representative of the entire population of participants (*N*), comparisons of the frequency distributions of “age class” in the different races were carried out using a one-sample Chi-squared test. The analysis showed that the sample was broadly representative of the overall population, with some relatively small (range: 0–4%) deviations showing that older women were slightly more likely to respond than younger. These deviations were statistically significant only in the main BR, though the achievement of a statistical significance might have been facilitated by the larger sample size. Information about this comparative analysis is provided in Table 2.

**TABLE 2:** Distribution of age and sex classification in population (*N*) vs. sample (*n*) for women participating in four Birkebeiner ski events.

**Class**	**Main race**		**Women-only 15 km**		**Women-only 30 km**	
**Age group**	***N* (%)**	***n* (%)**	***N* (%)**	***n* (%)**	***N* (%)**	***n* (%)**
<20	83 (6)	14 (3)	10 (1)	4 (1)	7 (1)	1 (1)
20–29	334 (23)	100 (20)	53 (7)	23 (6)	66 (13)	24 (13)
30–39	203 (14)	67 (13)	109 (14)	58 (16)	78 (16)	27 (14)
40–49	378 (26)	143 (28)	230 (29)	112 (31)	170 (34)	71 (38)
50–59	367 (25)	147 (29)	213 (27)	104 (29)	125 (25)	45 (24)
60–69	110 (7)	35 (7)	170 (22)	58 (16)	48 (10)	20 (11)
χ*^2^*	*15.76; p* = *008*		*8.11; p* > *0.05*		*0.65; p* > *0.05*	

*Age classification was not available for the Friday race.*

##### Analysis of the quantitative data

The survey data were first examined using descriptive statistics separately for each race (the main BR, Friday, and the women-only races 15 and 30 km), and then analyzed using an ML approach in order to detect the most representative variables that predict women’s participation in the different races. The ML approach differs from “classical” statistical approaches in the extent to which it is not primarily theory-driven. ML is a process that enables computer systems to progressively improve performance on a specific task without being explicitly programmed, and it can be used for data analysis purposes in order to develop statistical models with a high level of precision (Jordan and Mitchell, 2015). This process makes it possible to identify more accurately the predictors that have relevant impact on the dependent variable, as well as possible confounders, and thus lead to high levels of explained variance. This approach has been previously applied to the secondary analysis of survey data, including also a study of physical activity patterns and factors associated with them in the Norwegian population (Rossi and Calogiuri, 2018).

The dependent variable in our analysis was “Race” (i.e., the race variant in which the women participated). To reduce the imbalanced class distribution of the respondent frequencies in the different ski races, a binary dependent class was created aggregating the main BR with the Friday race (“full-distance races,” *n* = 612), and the two women-only races (*n* = 575). All parameters described in section “Measures” were run in the analysis. Answer alternatives “I don’t know/I’m not sure” were not included in the analyses (final *n* = 1,131).

A recursive feature elimination with cross-validation process (RFECV) based on logistic regression performed on 50% of the dataset (Ttrain) selected the most relevant variables able to discriminate women who participated in either ski race class (i.e., full-distance or women-only races). The importance of the features was assessed by a normalized Gini coefficient (G). On the Ttrain + RFECV, the hyper-parameter of three different classifier algorithms was obtained: logistic regression, decision tree classifier, and random forest classifier. The classifiers were validated with a threefold stratified cross-validation strategy on the remaining 50% of the dataset (Ttest + RFECV). Each cross-validation fold was made by preserving the percentage of samples for each class. Each sample in the dataset was tested once, using a model that was not fitted with that sample. The goodness of the classifiers was assessed by precision, recall, and F1-score (f1). Precision indicates the fraction of examples that the classifier correctly classifies over the number of all examples that the classifier assigns to that class. Recall indicates the ratio of examples of a given class correctly classified by the classifier, while f1 is the harmonic mean of precision and recall. Additionally, in order to assess the validity of the classifiers, we compared our predictive models with two baselines. Baseline B1 randomly assigned a class to an example by respecting the distribution of classes and Baseline B2 always assigned the majority class. All statistical analyses were performed using Python version 2.6. Significance level was set at *p* < 0.05. Table 3 shows the performance of the classifiers built on the base of the features selected in each dataset through the ML approach. Among the different models, LR showed the highest level of performance. In particular, LR correctly allocated 76% of the cases with a precision of 77%.

**TABLE 3:** Models metrics of classifiers.

**Model**	**Dataset**	**Precision**	**Recall**	**f1**
DT	Full-distance races (main and Friday races)	0.73	0.74	0.74
	Women-only races (women-only races 15/30 km)	0.72	0.71	0.71
RF	Full-distance races (main and Friday races)	0.76	0.76	0.76
	Women-only races (women-only races 15/30 km)	0.74	0.74	0.76
LR	Full-distance races (main and Friday races)	0.77	0.78	0.78
	Women-only races (women-only races 15/30 km)	0.77	0.76	0.76
B1	Full-distance races (main and Friday races)	0.52	1.00	0.68
	Women-only races (women-only races 15/30 km)	0.00	0.00	0.00
B2	Full-distance races (main and Friday races)	0.52	0.54	0.53
	Women-only races (women-only races 15/30 km)	0.49	0.48	0.48

*DT, decision tree classifier; RF, random forest classifier; LR, logistic regression; B1, comparison with baseline randomly assigning a class to an example by respecting the distribution of classes; B2, comparison with baseline assigning the majority class.*

##### Analysis of the qualitative data

The qualitative data was analyzed with the purpose of gaining further insight into the patterns in the quantitative data. Thus, the quantitative measurements of the women’s motives for participating and perceptions of the race were used to inform the categorization of the qualitative data. Accordingly, the data was analyzed grouping the two women-only races (*n* = 52) and the full-distance races (“full-distance races,” i.e., main BR and Friday race; *n* = 64). Discussion took place between two members of the team in order to arrive at a valid set of comments, which were then grouped into the categories used to present the quantitative results.

## Results

### Women’s Participation Trends

**FIGURE 1:**
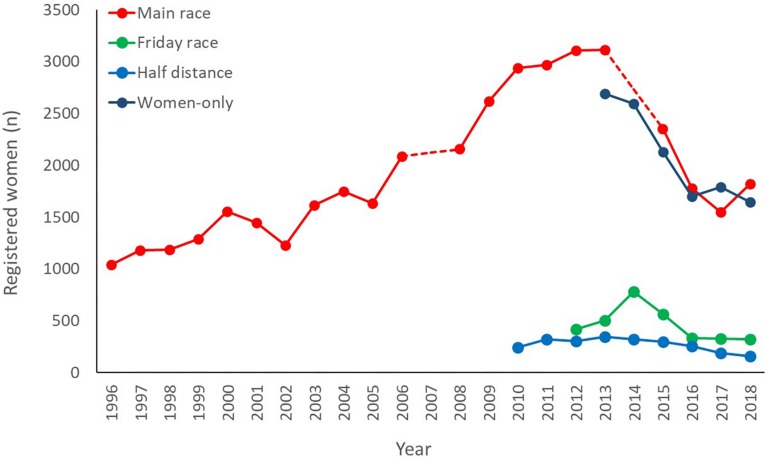
Entries for women (*n*), expressed as registered participants, in the main ski race and other races (Friday, half-distance, and women-only). The main race was canceled in 2007 and 2014, as marked by a discontinuous line. The women-only race came under the organization of Birken AS in 2013, thought it has been taking place since 1993 (entry records before 2014 are not available).

**FIGURE 2:**
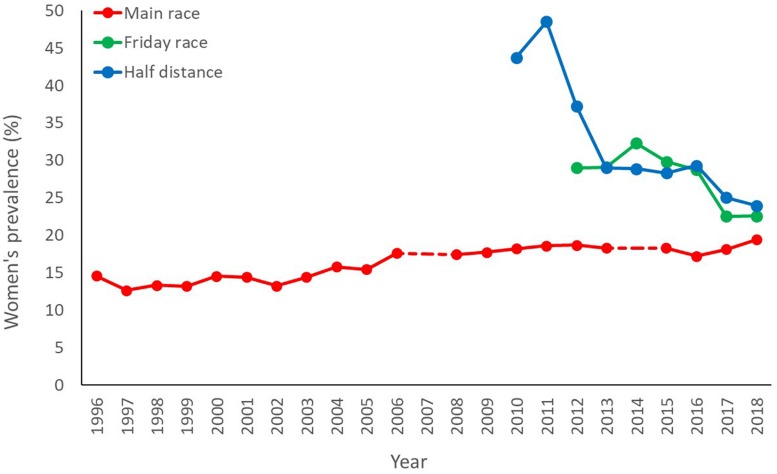
Prevalence of women in the main ski race, Friday race, and half-distance race, expressed as a percentage (%) of total participants registered. The main ski race was canceled in 2007 and 2014, as marked by a discontinuous line.

**FIGURE 3:**
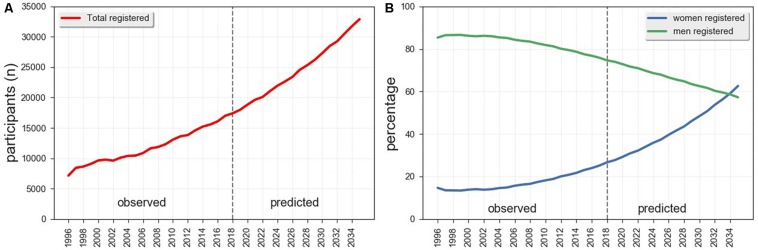
EWMAs for observed and predicted entries of **(A)** total participants and **(B)** percentage of men and women registering in the main race. The EWMAs are computed with a span of 6 years.

In the 17-year span between 1996 and 2013, the number of women participating in the main BR has trebled, though it dropped in the period 2015–2018 (Figure 1). It should be noted, however, that such fluctuation follows an overall trend in total participants. In spite of such fluctuations, the relative prevalence of women progressively increased from 13% in 1997 to 19% in 2018 (Figure 2). The autoregressive model confirms this trend (Figure 3), predicting a decrease in the percentage of male participants entries (MSE = 3.90%, residual error = 3.24 ± 2.17%) in favor of a relative increase in the women’s (MSE = 4.51%, residual error = 3.70 ± 2.58%). According to the outcomes of our model, if this trend persists, it would take about 15 years for the prevalence of women in the main BR to match the men. Moreover, the model predicts an increase in the total number of participants in the main BR for future years (MSE = 2,603 participants; residual error = 1,517 ± 254 participants), indicating that the number of women in the main BR is likely to increase both in relative and in absolute terms.

The introduction of the BR variants (Friday, the half-distance, and the women-only races) seems to have contributed to boosting women’s participation in the MPSE both in absolute and relative terms. In 2018, for example, the presence of the women-only races brought almost 1,600 women into the event, resulting in an increased overall prevalence of women from 20% to 30%. The presence of the women-only races also contributed to buffer the drop in women’s entries during the period 2015–2018. The Friday and the half-distance also contributed to boost the total number of women as well as their relative prevalence in the event. However, the relative prevalence of women in the Friday and the half-distance reduced progressively over the years: from 31% and 43% (respectively) in their first year of introduction, to 23% in 2018 (Figure 2). Thus, according to the latest records, the relative prevalence of women in the Friday races and half-distance race is only slightly larger than that registered in the main BR.

### Characteristics of Women Participating

Descriptive statistics for the sociodemographic characteristics, sport/exercise participation, and the psychological variables of women in the full-distance and women-only races are presented in Table 4. In general, most of the participants were middle-aged women with medium–high income from the most urbanized region of Norway (Oslo and Akershus). The event, however, also saw a relatively large participation of the women from the host region. A relatively large proportion of the participants (especially those in the full-distance races) reported to participate in other races, and most of them were planning to engage in at least one sporting activity in the 6 months following the race, with the most frequently reported activity being “running,” followed by biking and/or strengthening exercise. A large majority of women reported to have either maintained or increased their ski-based exercise habits compared with the past 5 years.

**TABLE 4:** Descriptive statistics and outcomes of the binary logistic model identifying the most important sociodemographic characteristics, exercise profile, and psychological factors that predict women participating in different Birkebeiner races in 2016.

**Participants’ characteristics**	**Full-distance races (*n* = 612)**	**Women-only races (*n* = 575)**	**Normalized Gini coefficient (%)**	***p***	**OR (95% C.I.)**
**Sociodemographic characteristics**					
Age (years), *M* ± SD	44.04 ± 11.75	46.21 ± 11.81	2	0.041	0.98 [0.97,1.00]
Income, *n* (%)			0	> 0.05	–
<200.000 NOK	71 (11)	37 (6)			
200–399.000 NOK	98 (16)	116 (20)			
400–499.000 NOK	252 (41)	256 (44)			
600–799.000 NOK	105 (17)	89 (15)			
800.000 + NOK	95 (15)	42 (7)			
Region, *n* (%)^a^			0	> 0.05	–
Host region	122 (19)	133 (23)			
Adjacent, most urbanized	261 (42)	228 (39)			
Other adjacent regions	132 (21)	162 (28)			
Farther regions	60 (9)	9 (1)			
**Sport and exercise participation**					
Other races, *n* (%)^b^	368 (60)	196 (34)	0	> 0.05	–
Ski-based exercise, *n* (%)			0	> 0.05	–
Decreased compared with 5 years ago	53 (8)	70 (12)			
Same as before	161 (26)	180 (31)			
Increased compared with 5 years ago	381 (62)	287 (49)			
*Type of exercise, n (%)*^b^					
Running	458 (74)	269 (46)	0	> 0.05	–
Biking	264 (43)	203 (35)	0	> 0.05	–
Roller-skiing	108 (17)	50 (8)	0	> 0.05	–
Organize sports	14 (2)	20 (3)	0	> 0.05	–
Strength exercise	211 (34)	227 (39)	0	> 0.05	–
Other	70 (11)	157 (27)	0	> 0.05	–
Sum of sports, *M*± SD	1.78 ± 0.89	1.7 ± 0.83	0	> 0.05	–
**Psychological variables**					
*Motives for participating, n (%)*^b^					
“It’s a motivational exercise goal”	487 (79)	351 (61)	0	> 0.05	–
“Because of the experience”	371 (60)	297 (51)	0	> 0.05	–
“It’s a nice nature experience”	284 (46)	227 (39)	0	> 0.05	–
“My friends did it”	59 (9)	235 (40)	36	0.001	5.94 [3.53,10.01]
“To improve my time from last year”	200 (32)	182 (31)	0	> 0.05	–
“I usually participate every year”	94 (15)	137 (23)	9	< 0.001	1.34 [1.16,1.54]
“To measure myself against others”	96 (15)	85 (14)	0	> 0.05	–
“It was a gift”	16 (2)	7 (1)	0	> 0.05	–
“It’s a work arrangement”	9 (1)	13 (2)	0	> 0.05	–
*Self-perception (1–6 scale), M* ± *SD*					
“Fit”	5.1 ± 0.97	4.89 ± 1.01	1	< 0.001	0.61 [0.47,0.79]
“Challenged”	4.95 ± 1.09	4.47 ± 1.27	0	> 0.05	–
“Part of a community”	4.56 ± 1.25	4.93 ± 1.08	6	< 0.001	1.50 [1.18,1.90]
“Capable”	4.34 ± 1.32	4.38 ± 1.25	0	> 0.05	–
“Extreme”	3.37 ± 1.43	2.43 ± 1.29	24	< 0.001	0.59 [0.51,0.70]
“Trendy”	3.14 ± 1.35	3.09 ± 1.46	0	> 0.05	–
“Ordinary”	3.12 ± 1.29	3.75 ± 1.36	0	> 0.05	–
*Race perceptions (1–6 scale), M* ± *SD*					
“A nice experience”	5.38 ± 0.80	5.49 ± 0.76	0	> 0.05	–
“The most important here is to complete against yourself”	5.20 ± 1.03	5.10 ± 1.21	0	> 0.05	–
“A nice nature experience”	5.12 ± 0.99	4.88 ± 1.16	0	> 0.05	–
“A good exercise goal for the season”	4.89 ± 1.37	4.33 ± 1.62	11	< 0.001	0.62 [0.52,0.74]
“A nice social atmosphere”	4.55 ± 1.38	4.88 ± 1.29	0	> 0.05	–
“An opportunity to compare myself with the best in my category”	3.59 ± 1.73	3.66 ± 1.72	0	> 0.05	–
“A tradition for me”	3.81 ± 1.85	4.6 ± 1.68	0	> 0.05	–
*Satisfaction and intention*					
Overall satisfaction (1–10 scale), *M* ± SD	8.11 ± 1.45	8.38 ± 1.56	12	< 0.001	1.50 [1.29,1.75]
Intend to participate next year, *n* (%)^b^	251 (41)	391 (68)	0	> 0.05	–

*Numeric variables are presented as means (*M*) and standard deviations (SD); categorical variables are presented as frequency (*n*) and percentage (%). ^*a*^Regions: *Host region* = Inland Norway (the region that hosts the races); *adjacent, most urbanized* = Oslo and Akershus (the most densely populated region, which is also better connected by road and rail to the rest of Norway); *other adjacent regions* = South-Eastern Norway (which are relatively close to the host region); *farther regions* = South-Western Norway, Western Norway, the Northern parts of central Norway, Northern Norway and abroad. ^*b*^Dummy variable or set of variables. Only the value for the “1” response alternative is shown, whereas the value for the “0” response alternative is not shown because redundant.*

For what concerns the psychological variables, “It’s a motivational exercise goal” was the most frequently reported participation motive across all races, followed by “Because of the experience” and “It’s a nice nature experience” or, for the women-only races, “My friends did it.” The most highly rated perceptions relative to self were “Fit” and “Challenged,” but also “Part of a community.” On the other hand, the most highly rated perceptions about the race were “A nice experience,” “The most important here is to complete against yourself,” and “A nice nature experience.” Levels of general satisfaction were remarkably high (mean value above eight for all races). Future participation intention was also relatively high, especially among the participants in the women-only races (68% reported it was very likely they will participate again the following year), while it was somewhat low among the participants in the main BR (41% reported it was very likely they will participate again the following year).

**FIGURE 4:**
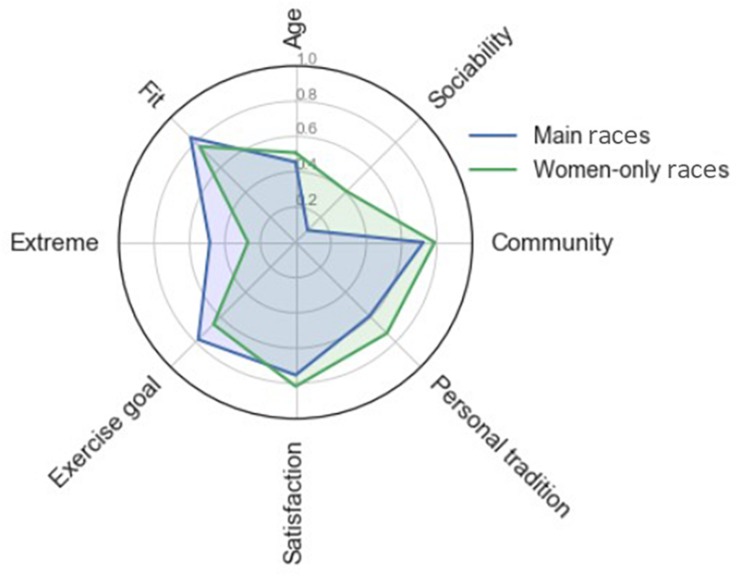
Radar chart of the features with highest explained variance (normalized Gini coefficient) in a logistic regression model predicting women’s participation in either the full-distance races (main race or Friday variant) or one of the two women-only races (15- or 30-km variants). Values expressed as the means of the questionnaire answers normalized to 0–1 range. Note: *Sociability* = “My friends did it” (participation motive); *Community* = “Part of a community” (self-perception); *Personal tradition* = “I usually participate every year” (participation motive); *Satisfaction* = Overall satisfaction with the race; *Exercise goal*: “A good exercise goal for the season” (race-perception); *Extreme* = “Extreme” (self-perception); *Fit* = “Fit” (self-perception).

The outputs of the logistic regression identified eight features as the most relevant predictors for the women participating either in the full-distance races (BR and Friday race) or the women-only races. Of the sociodemographic characteristics, only age was found to be a significant predictor, where older women were more likely to participate in the women-only races rather than the full-distance races (G = 0.02; OR [95% CI] = 0.98 [0.97–1.00]; *p* = 0.041). None of the sport/exercise participation variables were found to be significant predictors of women’s participation in either one of the two classes of races. The psychological variables had, on the other hand, a greater weight on the overall explained variance of the model. In particular, women who rated social aspects, such as feeling “part of a community” and reporting “sociability” (i.e., “my friends did it”) as an important participation motive, were more likely to participate in the women-only races rather than the full-distance races (G = 0.06 and 0.36; OR [95% CI] = 1.50 [1.18–1.90] and 5.94 [3.53–10.01]; *p* < 0.001 for both). Participation in the women-only races was also predicted by high ratings of overall satisfaction as well as perceiving the race as a “personal tradition” (respectively, G = 0.12 and 0.09; OR [95% CI] = 1.50 [1.29–1.75] and 1.34 [1.17–1.54]; *p* < 0.001 for both). On the other hand, women with greater perception of the race as “a good exercise goal for the season” (G = 0.11; OR [95% CI] = 0.62 [0.52–0.74]; *p* < 0.001) that makes them feel “extreme” and “fit” (respectively, G = 0.24 and 0.01; OR [95% CI] = 0.59 [0.50–0.70] and 0.61 [0.47–0.79]; *p* < 0.001 for both) were more likely to participate in the full-distance races rather than in the women-only races. The features selected by the LR model are summarized in the radar chart in Figure 4.

In line with “exercise goal” being a significant predictor for participation in the full-distance races, the qualitative data revealed that the full-distance races provided the participants with a challenge and hence the opportunity to “experience an enormous sense of mastery” (18 y.o.). This is further elaborated by the following quotation from an older participant describing how the demanding nature of the full-distance races contributed to her feeling of mastery: “… pushing myself, breaking barriers and achieving a sense of mastery” (28 y.o.). Additionally, it seemed that within the competitive context of the full-distance races, the sense of mastery extended to a sense of mastery over others: “I was challenged by colleagues” (age). For the participants in the women-only races, however, the discourse revolved more around “having fun” (44 y.o.). This seemed to be related to the terrain of the races; “the tracks are fun, with the hilly parts first, then the flatter parts afterward” (65 y.o.). Furthermore, it seemed as if the participants in the women-only races were more in competition with themselves, rather than their peers as illustrated by the following: “It’s a very ‘easy’ race to do without being in top shape, but you do get inspired to beat your own time!!” (54 y.o.).

While the quantitative data showed how sociability and community was a predictor for participation in the women-only races, participants of both races mentioned the general social atmosphere of the races as something that was meaningful to them. The difference between the full-distance and the women-only races was found in the kind of social connections experienced, where the full-distance races gave opportunities for the wider family to be involved and share the enjoyment of participating: “Me and my dad found we should try it out. My brother and partner joined as well, so it became a family thing” (31 y.o.). Moreover, the more challenging nature of the full-distance races seemed to provide more of an opportunity for family members to set themselves common goals: “I want to contribute to motivating my husband to get back on his feet after a serious accident in [year]” (32 y.o.). The women-only races on the other hand seemed to generate a dynamic social context for mother/daughter bonding and, in particular, for mothers to provide practical and social support: “my daughter competes, I follow as a ski waxer” (50 y.o.). The following quotation also illustrates how the women-only races provided opportunities for inter-generational sharing of enjoyable experiences with family members who were able to participate in different distances because of the way the races were organized:

“We have done all the women-only races, so it is tradition, and it is a nice experience that I share with my two daughters, one of which participates in the 30 km, while the other is finally enjoying the fact that she is beating her mother at 15 km” (55 y.o.).

Indeed, one respondent felt the race arrangers should emphasis the inter-generational possibilities of participation to a greater extent: “It is a race where mothers and daughters can participate together. It’s weird that there aren’t more people doing this, and that [the race arrangers] don’t highlight this better!”

When it comes to the significance of personal tradition, it seemed that the full-distance races mostly functioned as something of a “bucket list” thing; “A dream since the 70s” (55 y.o.). This seems especially pertinent given the iconic cultural status of the main BR in Norway. For the women-only races, the data suggest that it may have function in terms of having a life-long traditional purpose to it, locking participants into participating in the event: “I’ve done it all 24 times it has been arranged. So I just have to do it” (53 y.o.). In this way, participating in the race becomes a more or less embedded aspect of women’s lives even as they age, something that is made possible by the shorter distances and less demanding courses, but also desired because of the enjoyment experienced. One woman for example described how it had “been developed into a lifestyle the last 21 years” (73 y.o.).

## Discussion

### Women’s Participation Trends and Their Significance to Women’s Inclusion

In general, the findings show how women have been largely under-represented, compared to men, in the main BR as well as in the Friday and half-distance variants. This is consistent with research on MPSEs (Bowles et al., 2006; Andersen, 2019). However, some encouraging trends were observed. More specifically, in spite of some oscillations in the total number of women taking part in the main BR between 1996 and 2018, oscillations that mirror the trend of total participants in the race as well as general trends of skiing participation in Norway (Dalen and Holbaek-Hanssen, 2016; Statistics Norway, s.a.), the relative prevalence of women increased progressively. This trend is in line with analyses that indicate an increasing interest of women in major MPSEs (Andersen, 2019) as well as with trends in sports participation in the Norwegian population in general (Statistics Norway, 2015, s.a.). Moreover, our findings predict that the women’s relative prevalence in the main BR will continue increase in years to come, possibly with the gender split being completely overcome in about 15 years.

In terms of the impact of the other race variants (Friday race, half-distance race, and women-only races) in boosting women’s participation in this sporting event, the findings indicate that these races, and especially the women-only races, contributed to increasing the ratings of women both in absolute and relative terms. The women-only races, especially, contributed to buffering the drop among the participants in the main BR in the period 2015–2017, doubling the ratings of women’s entries in that same period. However, in contrast to the trends observed in the main BR race, the relative prevalence of women in the Friday race and half-distance race has been declining since the year of their introduction. This might suggest a “shift” in interest of the women toward the main (more competitive) race, though more research is warranted in order to corroborate such an assumption.

The introduction of women-only races (such as the Women-only BR in Norway and the Women’s Mini Marathon in Ireland, just to mention two) appears as an important and effective strategy to broaden the range of women participating in MPSEs. However, as Hausken (2019) notes from a physiological point of view, short distance races are unwarranted and even unethical as full-distance races suits the women’s physiology just as well as men’s. Thus, seen in line with the progressive increase in women’s participation in the main (full-distance) races, a trend confirmed in our study, there is a need for more efforts to foster greater inclusion of women in these events as well. It is indeed encouraging to notice that, in recent years, initiatives and programs have been launched to work to increase female participation in different types and at all levels of MPSE (see, e.g., Women For Tri^TM^ and The Women’s National Runner Survey from Running USA©).

### Participants’ Characteristics and Their Significance for Exercise Promotion

The sociodemographic profile of our sample, predominantly middle-aged women with medium–high income and living in the most urbanized region of Norway, is in line with known patterns in MPSEs participation (Murphy et al., 2015). On the other hand, the relatively large proportion of participants from the “host regions” (Inland Norway), which is remarkable especially considering the low-population density of this area, is of interest considering the poor public health profile of this area, especially in relation to low physical activity levels of its population. This finding suggests that MPSEs might have a particular beneficial impact in local communities. Arguably, this is somewhat in tension with the common approach of organizing MPSEs in major cities (see, for example, popular city marathons).

Our sample appeared to be constituted predominantly of “sporty” women, with many of them reporting their participation in similar competitions and planning to engage in at least one sporting activity during the spring season. This lends support to earlier findings indicating that MPSEs mainly reach those already adequately active (Lane et al., 2010, 2012). It is worthy of note, however, that about 60% of participants overall reported they had increased their ski-based exercise habits compared with the past 5 years, which may suggest that participation in the MPSE might have been, for some women, part of the process of increasing their physical activity levels. This interpretation is corroborated by the fact that the participation motive “It’s a motivational exercise goal” was highly frequent, a finding that also emphasizes the goal-directed nature of participating in the MPSE as a health behavior. Nevertheless, none of these variables was a significant predictor of participation either in the full-distance races or the women-only races, indicating that the two groups of women had similar profiles in terms of sport and exercise participation. On the other hand, the older age of the women in the women-only races, alongside the women’s reports of experiencing the race as a “tradition,” suggests that the women-only races might have a greater potential than the main BR to help women maintain higher PA levels with aging. Such an assumption is in line with previous research on women-only MPSEs (Crofts et al., 2012; McArthur et al., 2014).

Worth of notice is the high importance of the participants given to *autonomous* forms of motivation (i.e., motivations driven by the intrinsic desire of pursuing an activity for its own sake, rather than for external rewards such as winning a prize or esthetic benefits). This could be noticed in the participants reporting “Because of the experience” as main participation motive, as well as perceiving the race as “A nice experience” and that “The most important here is to complete against yourself.” Autonomous forms of motivations, as well as positive emotional experiences, are particularly desirable in relation to exercise (and health) promotion, as they have been previously reported to predict more stable exercise behavior in the long term (Kwan and Bryan, 2010; Teixeira et al., 2012). The importance given to nature experiences (see “it’s a nice nature experience,” which was highly rated both, as a participation motive and as a race-perception), which can also be seen as a factor that can elicit long-term adherence to exercise (Calogiuri and Chroni, 2014).

Taken altogether, these findings partly confirm concerns regarding the limited impact that MPSE can have in promoting health and exercise among women in the general population, although it also provides some additional insights into possible ways in which MPSEs might help *some* women to achieve and maintain their exercise goals.

### Distinctive Profile of Women Participating in Different Events

Both the analysis of the quantitative and the qualitative data indicated that while participation in the women-only races was predominantly driven by the sociability motives and seeing the race as a tradition, the women in the full-distance races were predominantly driven by a performance-oriented motivation, and showed a desire to compete and feel “fit” and “pushing boundaries.” Both groups of women perceived the race as enjoyable, although those in the women-only race tended to report higher ratings of satisfaction than the women in the full-distance races. Our findings are partly in line with previous research showing that sport participation, compared with other forms of exercise (e.g., exercising in the gym), is primarily driven by more autonomous forms of motivation, such as mastery, enjoyment, and sociability (Frederick and Ryan, 1993; Kilpatrick et al., 2005; Calogiuri and Elliott, 2017). These same motives were indeed found to be important for women to enter the main BR or one of its variants. At the same time, our study emphasizes how participation motives are also largely related to the specific context of sporting events, as, for example, that of the full-distance races (for which participation was predominantly characterized by mastery- and performance-oriented motives) compared to that of the women-only races (for which participation was predominantly characterized by sociability-oriented motives).

Independently of the different characteristics identified in women participating in different races (full-distances vs. women-only), it is noteworthy that the motivational profile of the women that has emerged in this study is largely in contrast with the way contemporary media tends to portray women in MPSEs. A recent critical analysis of how women are presented in sport and fitness advertising (including advertising for MPSEs) showed how most often emphasis is put on body-oriented messages, glorifying (or at times “girlifying”) extreme esthetic body ideals (Drake and Radford, 2018, 2019). Based on our findings, more effective (and empowering) advertising targeting potential participants in full-distance MPSEs might instead focus on the women’s performance and athletic achievements, e.g., through modeling-based messages stimulating vicarious mastery experiences. On the other hand, messages targeting potential participants in women-only events should emphasize the exhilarating social atmosphere of the race.

### Strengths and Limitations of the Study

This study examined the under-researched topic of women’s participation in MPSEs, with an emphasis on the characteristics that distinguish women participating in different variants of the race, thus contributing to filling a gap in the research literature. Our focus on the public health dimension of the phenomenon also contributes further understanding of the extent to which MPSEs can contribute to promoting health in the population as well as the ideal of “sports for all.” Our analysis is based on novel and relatively large microdata from Norway, which had a relatively large response rate (35%), especially when compared with previous studies that used similar recruiting approaches; for example, Funk et al. (2011) had an overall response rate of 19% and Lane et al. (2010) had an average response rate of 23%. Moreover, our sample was found to be reasonably representative of the population of interest, at least in relation to age-class distribution. Finally, the inclusion of qualitative information allowed us to explore the phenomenon in more depth than hitherto.

This study has, however, a number of limitations that are worthy of note. First, in the study of participation trends, while a relatively large number of waves were available for a time-series analysis of the main BR, a relatively smaller number of waves were available for the other races (Friday race, half-distance race, and women-only races). This made the performance of a time-series analysis impossible for these races and the analysis of the participation trends less reliable.

In relation to the second part of the study, this was based on a secondary analysis of routinely collected data which may have resulted in some confounding variables being overlooked—a common limitation with secondary analysis (Cheng and Phillips, 2014). Moreover, although we performed a comparative analysis of our sample with the study population (i.e., the total participants registered), which showed a reasonable comparable distribution of age groups, the sample may not have been fully representative of the population of participants.

The instrument used in the survey was not validated. Furthermore, the inconsistency of the instruments used across years and among difference race participants made it impossible to include multiple survey waves in the analysis. This would have allowed a larger sample size as well as a better understanding of how the participants’ characteristics changed alongside the changes in participation rates. Moreover, although the qualitative information in this study provided additional insight into the women’s motivations and perceptions of the event context, the quality of these data was rather limited in terms of depth and richness (e.g., compared with qualitative data obtained through in-depth interviews).

Using a secondary analysis approach also limited the possibility of purposefully collecting data in line with specific theoretical frameworks, such as the *self-determination theory* (SDT), a psychological theory of motivation that has previously been used in research on sport motivation (see, e.g., Frederick and Ryan, 1993; Kilpatrick et al., 2005) as well as in the context of MPSEs (Funk et al., 2011). SDT posits that feelings of autonomy over one’s behavior—the perception that one is competent enough to perform a behavior—and feelings of relatedness or personal connection, converge to support the development and enactment of motivations (Deci and Ryan, 1985). For example, “Extreme” and “Exercise goal” are both clearly related to autonomy (the former, suggesting mastery experiences, referring to satisfaction of competence needs, while the latter suggesting identified or integrated levels of autonomous motivation).

Finally, the analysis of sociodemographic, physical activity, and psychological factors only included female participants (comparing women participating in different races), while comparisons with men were not performed. The comparison with men was, in this particular paper, problematic for various reasons. First, we were primarily interested in understanding the characteristics of female participants, examining the extent to which this reflects ideals of “sport for all” (e.g., the extent to which women with lower sociodemographic status and lower physical activity levels were represented) as well as the characteristics of women participating in different races. A comparison with men might, however, reveal interesting aspects that have been missed in our analysis. Thus, it is recommended that future research attempts to make such comparisons.

### Recommendations for Future Research

The topic of women’s participation in MPSEs remains under-researched. Some academic studies exist, but large parts of the information are still provided by market surveys or reports produced by race organizers or other organizations (see, e.g., Running USA©). We recommend that future research seeks to enhance the quality of data collection, employing validated questionnaires (possibly informed by solid theoretical frameworks) and in-depth interviews. Studies including follow-up assessment of physical activity levels pre- and post-race are also needed, as well as studies investigating long-term engagement with MPSEs (i.e., regular participants). We also recommend that future research focuses on women’s perceptions of barriers and factors that might negatively affect the race experience.

## Conclusion

The results from this study shed light on women’s participation in an iconic MPSE in Norway. Considering that in Norway levels of sport participation among women are relatively high, and that cross-country skiing is (still) embedded in the national identity of the population, this context offers interesting insight into the larger topic of women’s participation in MPSEs. Specifically, our research offers insights into the role of event configuration in providing meaningful experiences to differing sub-groups of women. In general, the findings corroborate known patters in MPSEs, especially with respect to: (i) low involvement of women, as well as other disadvantaged sub-groups (e.g., women with lower socioeconomic status and low physical activity levels); (ii) indication of a progressively increasing prevalence of women in the main race; (iii) different sociodemographic and motivational profile of women engaged in different races (especially when comparing full-distance vs. women-only races). On the other hand, novel and encouraging findings have also been highlighted, such as a relatively large involvement of local communities, indicating some potential benefits in terms of exercise promotion in a region with higher prevalence of insufficiently active individuals (rather than, for example, to MPSEs taking places in major cities). Moreover, by focusing on women’s participation across different races, our findings show that the specific race context plays an important role in broadening and supporting women’s inclusion in sporting events. It is worthy of note, however, that the combination of main race and its variants seems to be a good strategy for locking people in to participation over time as they age and develop different interests. More research is needed in this field, which can help further enhance the understanding of how to best foster women’s participation in MPSEs.

## Data Availability Statement

The datasets generated for this study will not be made publicly available. The datasets used and/or analyzed during this study are of property of Birken AS (Rena, Norway) and Differ Strategy Consulting (Oslo, Norway). The datasets used and/or analyzed during the current study may be available from Differ Strategy Consulting on reasonable request.

## Ethics Statement

The studies involving human participants were reviewed and approved by the Norwegian Centre for Research Data (Project No. 58439). Written informed consent for participation was not required for this study in accordance with the national legislation and the institutional requirements.

## Author Contributions

GC conceived and designed the study, led the team of authors, and drafted the overall manuscript. PJ retrieved the data, structured the datasets and, together with MT, performed the analysis on the qualitative data and drafted the presentation of methods and findings relative to this part. AR performed the analysis on the quantitative data and drafted the presentation of methods and findings relative to this part. All authors provided substantial contributions to the design of the study as well as the revision of the intellectual content and final development of the manuscript and approved its final version.

## Conflict of Interest

The authors declare that the research was conducted in the absence of any commercial or financial relationships that could be construed as a potential conflict of interest. Birken AS designed the questionnaire used in the survey and sponsored the collection of data, which was performed through a private statistical agency (Differ Strategy Consulting, Oslo). Birken AS had no role in the design of the study, the analyses, the interpretation of the findings, or in the decision of writing the present paper. The authors are not employed at Birken AS or otherwise related to projects of this organization.
